# The utility and caveat of split-GAL4s in the study of neurodegeneration

**DOI:** 10.1080/19336934.2023.2192847

**Published:** 2023-03-23

**Authors:** Luca Stickley, Rafael Koch, Emi Nagoshi

**Affiliations:** Department of Genetics and Evolution and Institute of Genetics and Genomics of Geneva (iGE3), University of Geneva, Geneva, Switzerland

**Keywords:** Split-GAL4, neurodegeneration, LRRK2, Parkinson’s disease, dopaminergic neurons, PAM neurons

## Abstract

Parkinson’s disease (PD) is the second most common neurodegenerative disorder, afflicting over 1% of the population of age 60 y and above. The loss of dopaminergic (DA) neurons in the substantia nigra pars compacta (SNpc) is the primary cause of its characteristic motor symptoms. Studies using *Drosophila melanogaster* and other model systems have provided much insight into the pathogenesis of PD. However, little is known why certain cell types are selectively susceptible to degeneration in PD. Here, we describe an approach to identify vulnerable subpopulations of neurons in the genetic background linked to PD in *Drosophila*, using the split-GAL4 drivers that enable genetic manipulation of a small number of defined cell populations. We identify split-GAL4 lines that target neurons selectively vulnerable in a model of *leucine-rich repeat kinase 2* (*LRRK2*)-linked familial PD, demonstrating the utility of this approach. We also show an unexpected caveat of the split-GAL4 system in ageing-related research: an age-dependent increase in the number of GAL4-labelled cells.

## Introduction

*Drosophila melanogaster* has emerged as a powerful model system to study neurodegenerative disorders, owing to the conservation of genetic programmes and fundamental neurobiology between flies and humans, as well as its wealth of genetic tools [1]. A prime example is the research on Parkinson’s disease (PD), which is characterized by the loss of dopaminergic (DA) neurons in the substantia nigra pars compacta (SNpc) and the resulting locomotor impairments. DA neuron loss is frequently associated with the accumulation of intracytoplasmic inclusions mainly composed of the aggregates of α-synuclein protein, termed Lewy bodies (LBs), and those in the neurites, termed Lewy neurites (LNs) [2]. Discoveries of mutations linked to familial PD have led to the generation of numerous PD models in flies, which have made substantial contributions in understanding pathophysiology and pathogenic mechanisms of PD [3,4].

Understanding pathogenesis of PD requires the identification and characterization of vulnerable neuronal populations. While PD is increasingly recognized as a multisystem disorder, its pathology is observed nevertheless in restricted cell types. Postmortem analysis of PD patients tissues has shown the appearance of LBs and LNs in peripheral nervous system, including the enteric neurons and the autonomic nervous system, as well as in multiple regions of the brainstem [5]. The LB and LN pathology is also observed in neocortex in patients of advanced stages [6]. Notably, within the midbrain DA neuron population, the ventral tegmental area (VTA) and the retrorubral area (RRA) are affected to a much lesser extent than the SNpc [5], suggesting that cell-type difference has a non-negligible role in the pathogenesis. It remains unclear whether specific subtypes of SNpc DA neurons are selectively vulnerable in PD, although the advent of single-cell RNA-sequencing technology is closing this knowledge gap [7].

Here, we describe an approach to identify vulnerable subpopulations of DA neurons in the genetic background linked to PD in *Drosophila*. The method takes advantage of the split-GAL4 drivers, which enable the expression of transgenes in a small number of defined subsets of cells [8–10]. We show the utility of this powerful system by identifying a subtype of DA neurons selectively vulnerable in the model of the *leucine-rich repeat kinase 2* (*LRRK2*)-linked familial PD. We also show an unexpected and important caveat of this system for ageing-related research: the age-dependent increase in the number of cells labelled by the split-GAL4.

## Materials and methods

### *Drosophila* culture and strains

Flies were raised on standard cornmeal-agar food at 25°C in a 12 h:12 h light–dark cycle and under controlled humidity. Following fly strains were obtained from the Bloomington *Drosophila* stock centre (BDSC): *20×UAS-6×GFP* (Bl #52261), MB316B (Bl #68317), MB032B (Bl #68302), MB056B (Bl #68276), MB109B (Bl #68261), MB025B (Bl #68299), MB047B (Bl #68364), MB042B (Bl #68303) and MB188B (Bl #68268). *UAS-Lrrk*^*I1915T*^ was described previously [11,12].

### Immunohistochemistry

Immunostaining was performed as previously described in [11]. Briefly, fly heads were fully dissected and fixed in 400 µL of 4% paraformaldehyde (PFA), 0.3% Triton X-100 for 20 min on ice. Once fixation was completed, three quick washes followed by three 20-min washes were performed in PBS, 0.3% Triton X-100. Blocking was achieved with 1 h incubation in 5% normal goat serum (NGS), PBS, 0.3% Triton X-100 at room temperature on a rocking platform. Brains were incubated with the primary antibodies for two nights at 4°C on a rocking platform. Brains were then washed and incubated with the secondary antibodies overnight at 4°C. Vectashield (Vector Laboratories) was used as slide mounting medium. Antibodies used in this study and dilutions were as follows: Mouse anti-nc82 (mouse, Developmental Studies Hybridoma Bank, 1:100), Rabbit anti-GFP (G10362, Invitrogen. 1:500), Goat anti-Rabbit IgG Alexa Fluor 488 1:200 (A11008, Thermo Fisher, 1:200) and Goat anti-mouse IgG-Alexa Fluor 633 1:200 (mouse, A21052, Thermo Fisher, 1:200).

### Imaging and image analysis

Fly brains were scanned using Leica TCS SP5 confocal microscope, at 40× with 1.4× zoom for cell counting. 1.0× zoom was used for whole brain overview. Quantification of cell numbers was performed as described [13,14], using the cell counter plugin of Fiji (Fiji is just ImageJ) [15].

### Statistics and graphics

Statistics were performed in Python with the help of several statistical packages. Comparison between two conditions was performed with the ttest_ind function of the Python SciPy Stats package [16]. Two-tailed Student’s *t*-test was used to compare two groups. Groups with more than one factor were compared with ANOVA with a Tukey’s HSD post-hoc test using the pairwise_tukeyhsd function of the Python statsmodel package [17]. Statistical significance for all comparisons was set at *p* < 0.05. Graphical representation of data distributions was performed according to the guidelines of raincloud plots with the PtitPrince Python package [18].

## Results

We and others have previously shown the loss of dopaminergic neurons (DA) within the protocerebral anterior medial (PAM) cluster by genetic or pharmacological insults in adult *Drosophila* brains [11,13,14,19]. Although the number of PAM neuron loss observed so far varies between 15% and 80%, other DA clusters remain unaffected. These results are reminiscent of regionally restricted dopaminergic neurodegeneration described in early human PD cases, where substantia nigra pars compacta (SNpc) DA neurons are more susceptible to neurodegeneration than ventral tegmental area (VTA) DA neurons [20]. The underlying cause of this distinction between nuclei is unclear, but much of the discussion revolves around morphological as well as electrophysiological differences [20].

**Figure 1.**
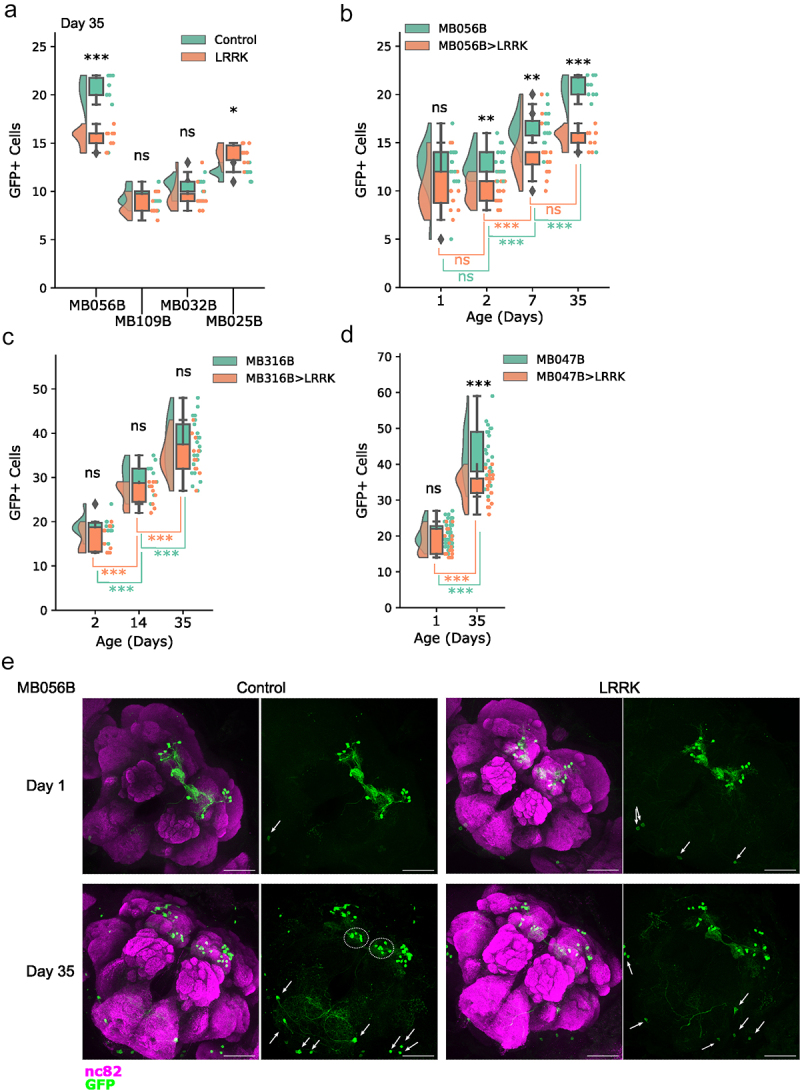
Split-GAL4 based approach to identify DA neurons susceptible to degeneration. (a). The number of GFP-positive cells in the flies expressing *GFP* with (LRRK) or without (Control) *Lrrk*^*11915T*^ driven by the indicated split-GAL4 drivers. Day 35. Each data point indicates the number of GFP-positive cell per hemisphere. Diamonds represent outliers. Tukey HSD test was used to compare the values between the control and LRRK groups of the same genotype. MB056B ***p = 0.001 (*n* = 10); MB109B *p* = 0.6941 (*n* = 10); MB032B *p* = 0.5872 (*n* = 10); MB025B *p = 0.0319 (*n* = 10). *Lrrk*^*11915T*^ driven with MB056B significantly reduced the number of GFP-positive neurons, whereas more GFP-positive neurons were observed in the LRRK group compared to the control with MB025B. ns, not significant. (b) The number of GFP-positive cells in MB056B control or MB056B> *Lrrk*^*11915T*^ at day 1, 2, 7 and 35. The graph shows progressive increase in the both the number of neurons and disparity between the genotypes. Tukey HSD test was used for comparing the control and LRRK groups for each time-point. Day 1 *p* = 0.0864 (*n* = 12–16), day 2 **p = 0.0011 (*n* = 20), day 14 **p = 0.0078 (*n* = 16), day 35 ***p = 0.001 (*n* = 10). Green lines, age comparisons within the control group. Tukey HSD; day 1–2 *p* = 1.0 (*n* = 16–20), day 2–7 ***p≤0.001 (*n* = 16–20), day 7–35 ***p≤0.001 (*n* = 10–16). Orange lines, age comparisons within the LRRK group. Tukey HSD; day 1–2 *p* = 1.0 (*n* = 12–20), ***day 2–7 *p*≤0.001 (*n* = 12–16), day 7–35 *p* = 0.4806 (*n* = 10–16). (c) The number of GFP-positive cells in MB316B control or MB316B >*Lrrk*^*I1915T*^ at day 2, 14 and 35. An increment in number of labelled neurons is also present in this split-GAL4. No comparison between control and *Lrrk*^*11915T*^ groups is significant. Tukey HSD; Day 2 *p* = 0.7347 (*n* = 10), day 14 *p* = 0.5731 (*n* = 10), day 35 *p* = 0.802 (*n* = 11–24). Green lines, age comparisons with the control group. Tukey HSD; day 2–14 ***p≤0.001 (*n* = 10), day 14–35 ***p≤0.001 (*n* = 10–24). Orange lines, age comparison within the LRRK group. Tukey HSD; day 2–14 ***p≤0.001 (*n* = 10), day 14–35 ***p≤0.001 (*n* = 11–24). (d) The number of GFP-positive cells in MB047B control or MB047B >*Lrrk*^*I1915T*^ at day 1 and 35. Tukey HSD test comparing the groups for each time-point. Day 1 *p* = 0.3212 (*n* = 20–46), day 35 ***p≤0.001 (*n* = 21–24). Green lines, age comparison within the control. Tukey HSD; day 1–35 ***p≤0.001 (*n* = 24–46). Orange lines, age comparison within the LRRK group. Tukey HSD; ***day 1–35 *p*≤0.001 (*n* = 20–21). (e) MB056B control and MB056B>*Lrrk*^*11915T*^ at day 1 and 35. Representative maximum intensity z-projections showing neuropil staining by anti-NC82 and MB056B-labelled cells with anti-GFP. Dotted circles indicate the cells that became more detectable in older flies. GFP-positive cells detected in the suboesophageal ganglion zone and near lobula (arrows) were not quantified. Scale bars = 50 μm.

To investigate potential morphological and electrophysiological differences between degenerating and non-degenerating DA neurons, we wanted to find and describe a susceptible PAM subpopulation. PAM neurons comprise approximately 20 subpopulations, projecting to different subdomains of the mushroom body (MB) [21]. Taking advantage of the Janelia FlyLight split-GAL4 drivers targeting different subpopulations, we sought to identify PAM neuron subtypes that are vulnerable in PD-linked genetic predispositions. To this end, we focused on a genetic model of *leucine-rich repeat kinase 2* (*LRRK2*)-linked familial PD, based on a targeted expression of a mutant form of *Drosophila Lrrk* (*Lrrk*^*11915T*^) [12]. The split-GAL4 lines function by using two promoter sequences, one regulating the expression of the DNA binding domain (DBD.GAL4), while the second the activator domain (AD.GAL4), which then combine to form a fully functional GAL4 [8]. We initially selected three lines targeting the PAM-β’2 subdomain (MB056B, MB109B and MB032B) and one line targeting PAM-β’1 (MB025B) (Table 1), because PAM neurons projecting to the MB β’ lobe are required for startle-induced locomotion, which declines with age and in various PD models [22]. In particular, the synaptic structure of the PAM neurons projecting to the MB β’ lobe progressively degenerates upon expression of human α-synuclein, leading to locomotor deficits [23]. Evaluation of the susceptibility was performed by expressing either *UAS-GFP* alone or *UAS-GFP* and *UAS- Lrrk*^*11915T*^ with each respective driver, followed by dissection at the age day 35 and counting of GFP-positive neurons in the PAM region. Expression of *Lrrk*^*11915T*^ with MB032B and MB109B caused no significant reduction in GFP-positive cell counts, while a significant decrease was observed with MB056B (Figures 1a–e). Curiously, MB025B-expressing cells were increased in number in the group expressing *Lrrk*^*11915T*^ (Figure 1a). These results suggest that MB056B positive but MB032B and MB109B negative cells, i.e. PAM-β‘2p neurons, are susceptible to LRRK2-induced neuronal loss.

**Table 1.** List of Split-GAL4 lines used and the PAM neuron subtypes targeted. (Ad.Gal4) Activator domain inserted at site attP40 (2 L) landing site, while DNA Binding Domain (Dbd.Gal4) uses the attP2 (3 L) landing site. Expression, PAM neuron subtypes targeted by each line, as described in [21].

Name	AD.Gal4 (attP40)	DBD.Gal4 (attP2)	Expression
MB056B	R76F05	R80G12	PAM-β’2 m, PAM-β’2p
MB109B	R76F05	R23C12	PAM-β’2a
MB032B	R30G08	ple	PAM-β’2 m
MB025B	R24E12	R52H01	PAM-β’1ap, PAM-β’1 m
MB316B	R58E02	R93G08	PAM-β’2 m,PAM-β’2p, PAM-γ4, PAM-γ4<γ1γ2
MB047B	R58E02	R93A09	PAM-β’2 m, PAM-β’2p
MB042B	R58E02	R22E04	PAM-β’2 m, PAM-γ3, PAM-γ4, PAM-γ5
MB188B	R58E02	R11A03	PAM-β’1ap, PAM-β’1 m, PAM-γ3, PAM-γ4

To further test the selective vulnerability of PAM-β‘2p neurons, we included two more split-GAL4 lines targeting this subpopulation, MB316B and MB047B (Table 1), in the following analyses. Since it is important to determine if loss of DA neurons is solely developmental or increases with age, GFP-positive neurons labelled by these drivers and MB056B, with or without co-expression of *Lrrk*^*11915T*^, were monitored at different ages up to day 35. GFP-positive cells located far from the PAM region (such as shown in Figure 1e, arrows) were not included in the quantification. MB056B-labelled cells observed at day 1 and 2 showed promising results, with clearer separation between the control and *Lrrk*^*11915T*^groups in 2-d-old flies (Figures 1b–e). This trend continued at timepoints day 7 and day 35, but of grave concern was the ever-increasing number of GFP-positive neurons in both control and *Lrrk*^*11915T*^ groups. Expression pattern of MB056B at day 1 was similar to that shown in the Janelia FlyLight website, mostly consisting of PAM-β‘2 subgroups. Whereas at day 35, numerous cells located more medially were additionally detected in the PAM region (Figure 1e, dotted circles). It was unclear which subpopulations were reduced in the presence of *Lrrk*^*11915*^.

The number of MB316B-labelled cells appeared to be reduced in the *Lrrk*^*11915T*^ group in older flies, but the differences were not statistically significant. The age-dependent increase in the number of GFP-positive cells was still present in both groups (Figure 1c). MB047B-labelled population increased in number from day 1 to 35, with a significant reduction in the *Lrrk*^*11915T*^group at day 35 compared to the control (Figure 1d). These results support that susceptible neurons belong to the subpopulation targeted by MB056B and MB047B. The data also suggest that MB316B expression only partly covers susceptible neurons. However, due to the broadening of GFP-positive cells by age, we were unable to determine at what age these cells were lost and which exact subpopulation they belonged to.

**Figure 2.**
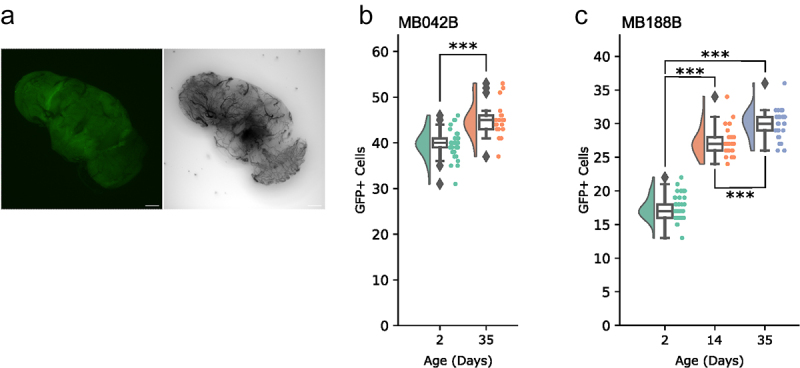
Incremental labelling by split-GAL4s.

The strain carrying only the *UAS-GFP* transgene had no discernible GFP signal in the brain (Figure 2a). Concerned about the generality of the observation, we examined expression of two more split-GAL4 lines, MB042B and MB188B, which do not target PAM-β‘2p neurons (Table 1), at different ages. Age-dependent increment in GFP-labelled cells was observed in both lines (Figure 2b,c). Therefore, the increase in target population by age may be a general issue with split-GAL4s, which unfortunately makes this approach suboptimal for the study of age-dependent loss of DA neurons.

(a) A representative image of the brains of *20*x*UAS-6×GFP* flies, showing no specific GFP signal throughout the brain. 8 d old. Scale bars = 50 μm. (b and c) Quantification of GFP-positive cells in the flies expressing GFP with MB042B (b) and MB188B (c) at indicated ages. MB042B, Student’s *t*-test; day 2–35 ***p≤0.001 (n = 19–34). MB188B, Tukey HSD; day 2–14 ***p≤0.001, day 2–35 ***p≤0.001, day 14–35 ***p≤0.001 (n = 28–51).

## Discussion

### Split-GAL4 incremental labelling

The number of cells expressing GFP driven by several split-GAL4 drivers increases by age and does not plateau even between days 7 or 14 and 35 of age. Whereas classical GAL4s are expressed monolithically, split-GAL4s require the activator domain (AD.Gal4) and the DNA-binding domain (DBD.Gal4) to combine and perform its function. This is achieved by placing a leucine zipper (Zip) on a flexible linker at the N-terminus of Gal4-DBD.Gal4 (Zip-) and the C-terminus of p65-AD.Gal4 (Zip+) [8,9]. Although age-dependent increase in the activity of one or both of the promoters expressing the split-GAL4 components could result in the incremental GAL4 reconstitution, it seems unlikely that activity of involved promoters does not reach a plateau until day 35, if not impossible. As MB056B and MB047B do not share either AD.Gal4 or DBD.Gal4 promoters, such a mechanism would be independent of the inserted sequences, for example, via changes in chromatin accessibility by age. Instead, low turnover of reconstituted GAL4 or GAL4-promoter complex may be a key contributor of the incremental labelling. The binding of monolithic GAL4 to the promoter has a short half-life *in vivo* [24]. It is worth investigating if reconstitution of GAL4 by heterodimeric leucine zippers increases GAL4 stability or changes turnover rate of GAL4-promoter binding. This feature, if indeed the case, would be an advantage in some applications.

This study examined only five split-GAL4 lines for age-dependent expression changes, and it remains to be seen whether the finding is generalized. We used hexameric GFP encoded by *20×UAS-6×GFP* [25] as a reporter and anti-GFP polyclonal antibodies for maximal detectability throughout this study. Since hexameric GFP is expressed from a single open reading frame and produced as a single polypeptide encoding six monomers of GFP fused in tandem, there is no theoretical reason that it should cause age-dependent increase in signal intensity. Nevertheless, it is important to test split-GAL4 expression with different reporters before establishing whether age-dependent broadening of target population is a general issue with the split-GAL4 system.

Of note, some split-GAL4 lines have ectopic expression in the regions far from the PAM cluster and the MB. This finding is in line with previous studies [9,26]. These ectopic cells also appear to increase in number by age. The ectopic cells that were located far from the MB were not included in our analysis as they were easily distinguishable from PAM neurons. However, the bigger problem is the unannotated cells that are found near the MB and become visible only in aged flies. In summary, our results call for caution in using the split-GAL4 system. When using split-GAL4s, the comparison between groups at the same age will be legitimate, whereas comparisons between different age groups would not produce meaningful results.

### Functional and morphological characteristics of vulnerable cell types

Although the artefact of split-GAL4 system did not allow continued investigations of this topic, the finding of possible functional susceptibility is still of note. We found that MB056B and MB047B lines, which target PAM-β’2 m and PAM-β’2p neurons, but not MB032B targeting PAM-β’2 m, mark the cells lost in the presence of *Lrrk*^*11915T*.^. As discussed above, ectopic GFP-positive cells near the PAM cluster were observed in older flies, which were included in the quantification. Nevertheless, the fact that two independent split-GAL4s constructed with entirely different promoters (Table 1) target vulnerable cells points to the possibility that susceptible cells are part of or those clonally related to the PAM-β’2p subgroup. Ninety per cent of DA neurons that project to the MB receive feedback of the MB output neurons (MBON) in the convergent zones (crepine, superior medial protocerebrum, superior intermediate protocerebrum and superior lateral protocerebrum), which in turn receive dendrites from the central complex, a region crucial for motor actions [21]. Scaplen et al. [27] has recently shown that the MBON- β‘2mp and MBON-γ5β‘2a have the highest number of projections to PAM neurons, while MBON-β 1 had one of the lowest. Although it is unclear if the PAM neurons synapsed by these MBONs are the same that innervate them, the differences in the PAM-MB-MBON-PAM feedback loop could explain the possibly increased loss of neurons in the PAM-β2p subgroup.

Work by Zhao et al. [28] has shown that *LRRK2* differs in its interactome depending on tissue, with clear shared features found in the putamen, caudate and nucleus accumbens. This finding encourages the studies of PAM-β‘2p susceptibility even with the hindrance of incremental labelling by split-GAL4s. Future work on selectively susceptible neurons will require more precise identification of affected cell types, for which MB056B and MB047B may prove useful.

## Data Availability

All the data are presented in the paper. The original image data are available upon request.
